# Biomarkers for Lysosomal Storage Disorders with an Emphasis on Mass Spectrometry

**DOI:** 10.3390/ijms21082704

**Published:** 2020-04-14

**Authors:** Ryuichi Mashima, Torayuki Okuyama, Mari Ohira

**Affiliations:** Department of Clinical Laboratory Medicine, National Center for Child Health and Development, 2-10-1 Okura, Setagaya-ku, Tokyo 157-8535, Japan; okuyama-t@ncchd.go.jp (T.O.); ohira-m@ncchd.go.jp (M.O.)

**Keywords:** Lysosomal storage disorders, biomarkers, mass spectrometry, enzyme activity, newborn screening

## Abstract

Lysosomal storage disorders (LSDs) are characterized by an accumulation of various substances, such as sphingolipids, mucopolysaccharides, and oligosaccharides. The LSD enzymes responsible for the catabolism are active at acidic pH in the lysosomal compartment. In addition to the classically established lysosomal degradation biochemistry, recent data have suggested that lysosome plays a key role in the autophagy where the fusion of autophagosome and lysosome facilitates the degradation of amino acids. A failure in the lysosomal function leads to a variety of manifestations, including neurovisceral disorders. While affected individuals appear to be normal at birth, they gradually become symptomatic in childhood. Biomarkers for each condition have been well-documented and their proper selection helps to perform accurate clinical diagnoses. Based on the natural history of disorders, it is now evident that the existing treatment becomes most effective when initiated during presymptomatic period. Neonatal screening provides such a platform for inborn error of metabolism in general and is now expanding to LSDs as well. These are implemented in some areas and countries, including Taiwan and the U.S. In this short review, we will discuss several issues on some selected biomarkers for LSDs involving Fabry, Niemann–Pick disease type C, mucopolysaccharidosis, and oligosaccharidosis, with a focus on mass spectrometry application to biomarker discovery and detection.

## 1. Introduction

**Figure 1:**
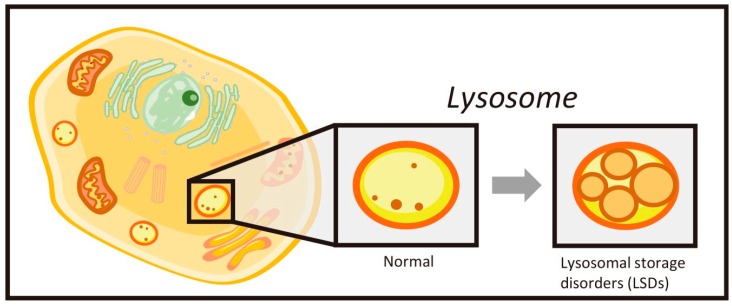
Cellular localization of lysosomes. Under normal conditions, the lysosome catabolizes sphingolipids, oligosaccharides, mucopolysaccharides, and other biological substances at acidic pH. In the lysosomal storage disorders (LSDs), such a compartment becomes enlarged and many cellular vacuoles are identified using ultramicroscopic technique in the lysosomes. When the process of autophagy is activated, the lysosome fuses to the autophagosome followed by enhancing the degrading process by lysosomal enzymes.

Lysosomal storage disorders (LSDs) are caused by a deficiency of lysosomal enzymes associated with an accumulation of substances, such as sphingolipids, mucopolysaccharides, and oligosaccharides (Figure 1) [1,2]. Although these are rare disorders, several therapies are now available to treat them. Enzyme replacement therapy (ERT) is an established treatment that infuses recombinant therapeutic enzymes in the body. Evidence has now proven that ERT is effective for visceral manifestations, while treatment of central nervous system (CNS) manifestations continues to be a major challenge. The reason largely relates to the blood–brain barrier (BBB) of endothelial cells which separates blood and brain. To gain more effective outcomes in CNS involvement using ERT, a novel enzyme agent generated by a polypeptide containing a monoclonal antibody against human transferrin receptors fused to therapeutic enzymes has been developed [3]. Combined with this therapeutic approach, an understanding of the natural history of LSD, often available through affected siblings, also plays a key role. Based on this experience, the best treatment outcomes are associated with its initiation during an asymptomatic period.

Neonatal screening is part of a public health program that is initially implemented in phenylketouria followed by expansion to LSDs. The best known example of the benefits of LSD screening programs comes from Taiwan, where the survival rate of infantile-onset Pompe disease has dramatically improved through ERT for affected individuals identified during neonatal screening by fluorometric detection [4]. Most LSD enzymes are active on dried blood spots (DBSs) during proper storage conditions. An earlier program for newborn LSD screening in Taiwan used fluorimetric detection from DBSs for a limited number of disorders such as Pompe, Fabry, and MPS I [5]. However, with an increasing number of disorders to screen for, it became clear that a single DBS-mediated multiplex assay has been accepted as beneficial due to the rarity of each condition. Currently, newborn screening for diagnosis of LSDs can involve either fluorometric assay (for enzymes) or mass spectrometry for enzymes or biomarkers, and the methodology of choice will vary depending on geographical prevalence, government policy and available technology.

**Figure 2:**
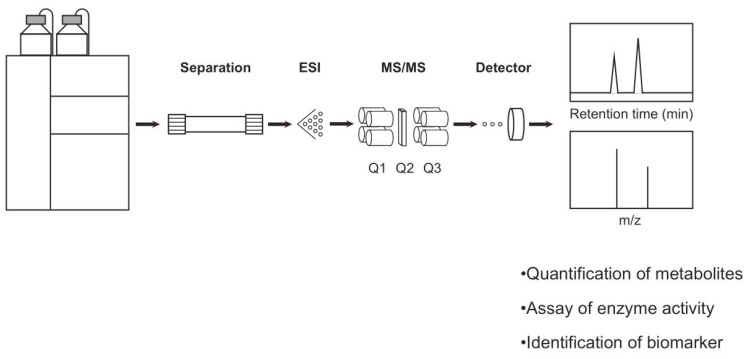
Representation of liquid chromatography with tandem mass spectrometry (LC-MS/MS). The analyte is injected onto an analytical column by a binary pump followed by separation with a variety of chromatographic technique, such as reversed phase and hydrophilic interaction chromatography. Then, eluate is ionized by an electrospray (ESI) technique. A droplet containing analyte was charged at the end of capillary followed by an introduction into a tandem mass spectrometer consisting of the first quadrupole (Q1), a collision chamber (Q2), and the second quadruple (Q3). The selected ion by a combination of each m/z for Q1 and Q3, respectively, is finally monitored by a detector. Multiple reaction monitoring (MRM) refers a technique that allows us to monitor multiple combination of Q1 and Q3.

Biomarkers for LSDs have been identified in many conditions. Primary biomarkers include either the substrate or product of enzyme reaction. For example, globotriaosylceramide (Gb_3_) is the substrate for α-galactosidase A (GLA), the enzyme responsible for Fabry disease. Heparan sulfate (HS) is one of mucopolysaccharide that elevates under pathogenic conditions in mucopolysaccharidosis type I, II, IIIA-D, and VII. Oligosaccharides are biomarkers for α/β-mannosidases as well as Pompe disease where glucose tetrasaccharide is elevated in some cases. All of these are elevated due to the deficiency of downstream enzyme activity. Classically, Gb_3_ has been detected using thin-layer chromatography with colorimetric detection. HS is usually separated from chondroitin sulfate, dermatan sulfate, as well as keratan sulfate by electrophoresis followed by detection using cationic dye including methyl methylene blue. Furthermore, the accumulation of oligosaccharide has been similarly shown by a combination of chromatographic technique and colorimetric detection. These previously established assays are now transferred to a renovated assay methodology such as high-performance liquid chromatography with tandem mass spectrometry (LC-MS/MS) (Figure 2). The latter has a higher sensitivity that requires much smaller amount of biological specimen.

**Figure 3:**
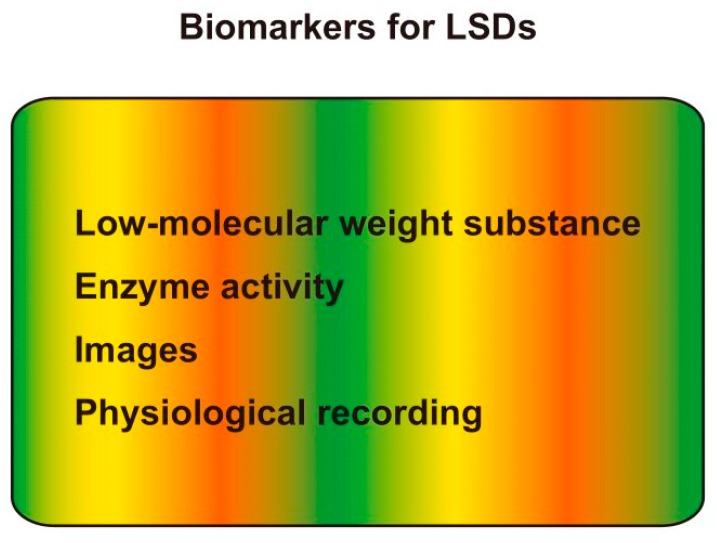
Biomarkers for lysosomal storage disorders (LSDs). The term ‘biomarker’ is not limited to low molecular weight compound that links to the elevation under pathophysiological settings and the reduction in response to treatment. It also covers imaging, physiological recording, as well as enzyme activity. The detail was discussed in the text.

The term ‘biomarker’ is often defined as a measure that correlates to the severity of the disorder or the response to pharmacological agents [6]. In this regard, an elevation of substances with low molecular weight appears to be most suitable for this definition (Figure 3). It is well accepted that the alteration of MRI imaging of the brain suggests some pathological events, thus this can be another biomarker. Physiological recording such as echocardiogram show the condition of heart, thus any abnormal sign will be linked to pathological conditions. Thus, additionally, it is no surprise that enzyme activity is included in this definition as a biomarker in clinical chemistry. Interestingly, although many clinically “good” biomarkers are well-defined, the other characteristics, such as biosynthesis, may not be fully described. Current trends for biomarker research include: (1) discovery of biomarkers with high specificity for particular disorders or disease subtypes, and (2) development of multiplex quantification methods for a group of biomarkers in sphingolipidoses and oligosaccharidoses. Apparently, metabolite-wide omics studies are deeply involved in the discovery for novel biomarkers. In the field of LSD, lipidomics, glycomics, proteomics, and genomics are related. Once any target biomarker has identified, then the levels of biological samples under pathophysiological settings are to be investigated. It is also important that assay should be robust, otherwise diagnosis become inconsistent. In this short paper, the authors will review recent publications that relate to these topics.

## 2. LysoGb_3_

**Figure 4:**
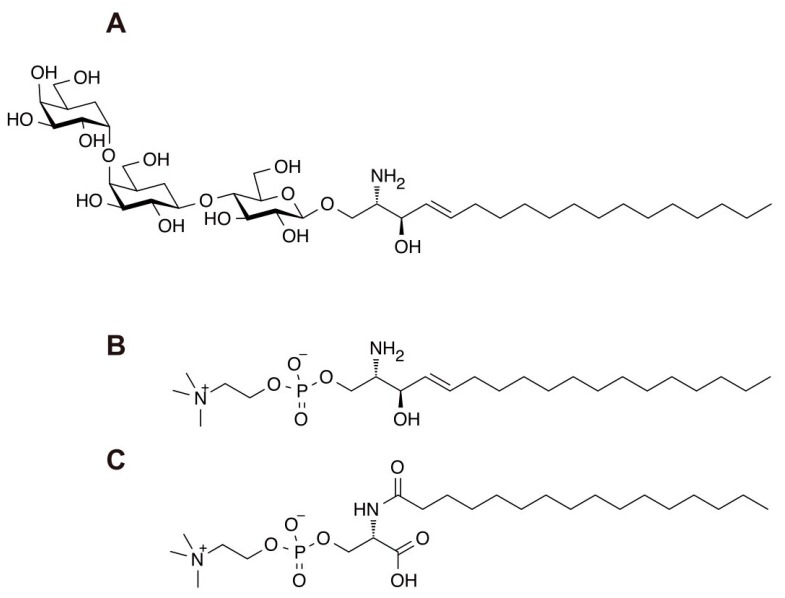
Structure of biomarkers for Fabry disease (**A**) and Niemann–Pick disease type C (**B**,**C**). (**A**) globotriaosylsphingosine (LysoGb_3_); (**B**) lysosphingomyelin (LysoSM); (**C**) *N*-palmitoyl-*O*-phosphocholine serine (PPCS).

Fabry disease is caused by a pathogenic mutation in the *GLA* gene [7]. Elevated Gb_3_, detected by thin layer chromatography, is a classical biomarker for Fabry disease. However, the metabolite LysoGb_3_ was discovered in 2008 as more accurate biomarker of disease manifestation and progression [8] (Figure 4A). LysoGb_3_ has hence become established as a preferred biomarker for Fabry disease. Based on extensive research, it is now known that healthy individuals contain less than 1 nM of LysoGb_3_ in their plasma, but classic and late-onset patients have two and one magnitude of order higher concentrations [9]. Furthermore, accumulating evidence indicates that a substantial number of females with Fabry disease have been identified [9,10,11,12]. In these cases, the pathogenic mechanism involves X-chromosomal inactivation where wild-type allele containing GLA gene is inactive. The concentration of plasma LysoGb_3_ in Fabry females may vary between normal concentrations (ie less than 1 nM) to similar concentrations found in classic Fabry males. Since Fabry females may not have a non-pathogenic allele, the diagnosis entirely depends on biomarkers and clinical manifestations. Quantification of LysoGb_3_ is best performed using ultra performance liquid chromatography-tandem mass spectrometry (UPLC-MS/MS) (Figure 2) with an internal standard as discussed later in this section. 

Emerging evidence demonstrates that LysoGb_3_ detected in the urine and plasma contains some variant substances [13]. Although the biosynthesis mechanism of LysoGb_3_ remains to be fully characterized, it is commonly accepted that LysoGb_3_ is generated from the enzymatic reaction of serine:palmitoyl transferase, a rate-limiting enzyme for sphingolipid biosynthesis [14]. In this case, long chain base (LCB), a building block of sphingolipids, is d18:1 representing a C18 aliphatic lipid with a double bond between C4-C5 with trans geometry and two hydroxyl groups. An extensive study revealed that plasma LysoGb_3_ contains small amounts of d16:1 and other species [15,16]. Apart from LCB, Fabry Gb_3_ contains a methylated form of *N*-acyl fatty acid. The concentrations in these types reach nearly the same concentration as the authentic Gb_3_. As we have been made aware, Fabry disease cases have been classified with classic and late-onset form. The latter is further subdivided into three subtypes: renal, cardiac, and CNS diseases [7]. Several groups reported that some of these Gb_3_ isomers could relate to any of the disease subtypes. These studies could establish disease-specific biomarkers for Fabry disease. Thus, the outcome of such a study will be useful for diagnosis, particularly for female patients where genetic testing will not provide any conclusive diagnostic evidence. A current challenge for the quantification of LysoGb_3_ includes the limited availability of proper internal standards. In principle, the electrospray ionization technique used for most commercially available LC-MS/MS instruments requires a very small droplet of organic solvent containing LysoGb_3_ and an internal standard for MS/MS detection. This ionization mechanism does increase the sensitivity of the analyte for smaller particle sizes but may lose quantification accuracy. The requirement for a suitable internal standard is currently problematic because of limited commercial availability associated with increasing production costs. Hong X et al. found that the α-1,4-galactosyltransferase C-mediated enzymatic reaction efficiently generated [^13^C]_6_-labeled LysoGb_3_ in the presence of [^13^C]_6_-UDP, which opens a promising new synthetic route for generating this internal standard the compound [17].

## 3. *N*-palmitoyl-*O*-phosphocholineserine (PPCS)/LysoSM-509

Niemann–Pick disease type C (NPC) is caused by a deficiency in either the *NPC1* or *NPC2* gene, the former shares 95% with pathogenic mutations worldwide [18]. A deficiency in these genes leads to an accumulation of cholesterol in the late endosome/lysosome, resulting in a variety of manifestations including CNS involvement, hepatosplenomegaly, and cataplexis. For diagnosis, an accumulation of cholesterol in the fibroblast from an affected individual has been used as established diagnostic evidence. In addition to cholesterol, there are many biomarkers associated with NPC. Oxysterols, namely 7-ketocholesterol and 3β,5α,6β-cholestane-triol, are useful biomarkers for NPC examined using LC-MS and gas chromatography-mass spectrometry (GC-MS). Second, lysosphingomyelin (LysoSM) is another NPC biomarker with unknown biosynthetic transformation that also correlates with NPC (Figure 4B). Third, a group of bile acids play a key role for diagnosing NPC. A seminal observation made by Alvelius [19] in a GC-MS assay opened an initial key to this field. Subsequently, a series of Japanese studies identified several bile acids as unique NPC biomarkers [20,21]. During the course of the studies, they found an interesting observation that some NPC-affected individuals may not have an elevating bile acid biomarker. This was finally rationalized by the homozygous deficiency of a UDP glycosyltransferase family 3 member A1 gene in a Japanese population [22]. Recently, *N*-(3β,5α,6β-trihydroxy-cholestan-24-oyl)glycine, known as bile acid B, has been added as a new biomarker for NPC [23,24].

LysoSM-509 was first reported in 2015 as being detectable by a combination of MS1/MS2 for m/z 509/184 with an electrospray ionization mode with positive polarity (Figure 4B) [25]. In the field of lipid biology, choline moiety in phosphatidylcholine and sphingomyelin is known to be readily detectable by a fragmentation ion m/z 184. Thus, it was quite reasonable that this putative biomarker has been named as a LysoSM-derivative with m/z value of MS1. Intriguingly, acid sphingomyelin deficiency causes an elevation of LysoSM-509 in the plasma [25,26,27]. Since this discovery, several groups tried to elucidate the chemical nature of LysoSM-509. In 2019, a U.S. and a Japanese group independently identified its chemical structure as *N*-palmitoyl-*O*-phosphocholineserine (PPCS) (Figure 4C) [28,29]. Both studies used chemical derivatization of LysoSM-509 followed by the synthesis of authentic chemicals including deuterated PPCS for LysoSM-509. Different species of *N*-acryl analog, including C14-C24, were elevated in human plasma. Although PPCS in NPC1-affected individuals was elevated significantly in the plasma, the level of PPCS in DBSs from NPC1-affected individuals was only two- to three-fold above controls. Notably, with the DBSs, the populations of NPC1-affected individual and healthy controls partly overlapped. Another interesting observation includes a marked animal specificity of PPCS accumulation. A possible explanation is that PPCS was generated during the drying process, reducing the sensitivity of DBS testing for this biomarker. PPCS was only detectable in humans and cats, but not in mice [28]. For unknown reasons, PPCS was generated during the drying process of DBS. Apparently, the biosynthesis of PPCS needs to be explored in terms of identification of precursor substances, metabolizing enzymes in specific animal species, and its physiological role in humans.

## 4. Heparan Sulfate

Mucopolysaccharidosis (MPS) is characterized by an accumulation of mucopolysaccharide including heparan sulfate, dermatan sulfate, chondroitin sulfate, and keratan sulfate. The concentration of mucopolysaccharide has been quantified as a complex of 1,9-dimethyl-methyleneblue (Reviewed in [30]). Later, the principle of quantification by binding anionic glycosaminoglycans with cationic dye was further applied to the detection method for electrophoresis. Thus, HS is quantified after fractionation from other glycosaminoglycan species, such as chondroitin sulfate, dermatan sulfate, and keratan sulfate, respectively.

Heparan sulfate (HS) is a biomarker for some disease subsets of MPS, including MPS I, II, IIIA-D, and VII, respectively [31]. Although the diagnosis of MPS may be possible by elevating HS in specimen, this evidence alone may not allow to identify the pathogenic gene. Thus, there has been a strong demand for the specific biomarker for each MPS disease subtype involving HS elevation. The reason behind this is attributed to an elevation of HS in all MPS III subtypes (i.e., MPS IIIA-D), as well as MPS I, II, and VII. Recently, Fuller and co-workers reported a panel of such biomarkers for MPS [31]. The quantification method involves derivatization of glycosaminoglycan (GAG)-derived urinary oligosaccharides with Michael addition reaction followed by reversed-phase LC-MS/MS. Similar to previous studies, elevated biomarkers for MPS I and MPS VI, such as UA-HNAc and HNAc, respectively, decreased significantly in response to ERT. They also showed that each disease subtype of MPS III, namely MPS IIIA-D, has a unique biomarker as listed in Table 1 [31]. 

Methanolysis is an established technique in glycan biology to estimate the distribution of monomeric saccharides, but is also applicable to the quantification of HS in the clinical specimen [32]. The optimized conditions may vary depending on the target saccharides. Typically, harsh conditions, such as acidic conditions, may be selected to maximize the yield of stable monosaccharides, while such reaction conditions may lose heat-labile saccharides such as sialic acid. Methanolic hydrochloric acid is a widely used reagent for methylation of carboxylic acid prior to the application of the gas chromatographic technique. It is known that all sulfate groups in glycosaminoglycans are lost during methanolysis [32]. When HS levels in a clinical specimen are to be quantified, chromatographic separation based on either hydrophilic-interaction chromatography [32,33,34] or reversed phase chromatography [35,36] was adopted. The optimal reaction period for heparan sulfate is not exactly same as that for other varieties such as chondroitin sulfate and dermatan sulfate [32]. Recently, an Australian group reported the synthesis of 12 species of potential HS methanolysates to understand which substance was mainly generated during this process [36]. They found that GlcN-(1→4)-uronic acid disaccharide was the major reaction product of HS methanolysis; in fact, the same conclusion was previously obtained by another study [37]. The authors also argued that butanolysis, rather than methanolysis, provides a much higher yield of methanolysate partly because of higher reaction temperatures.

When HS accumulation is going to be examined after enzyme assay has been performed, any assay for HS provides diagnostic information. This especially occurs when assay for HS is to be selected at the second tier of neonatal screening. For example, methanolytic HS concentration was quantified in Taiwan as the second tier testing [35].

## 5. Oligosaccharides

An accumulation of oligosaccharides has been known in some LSDs, such as mannosidosis, fucosidosis, and aspartylglucosaminuria. Due to a relatively larger molecular weight, which might be beyond the typical working range of a tandem mass spectrometer, these oligosaccharides are often identified using time-of-flight mass spectrometry (TOF-MS). The biological source of oligosaccharides may be derived from either *O*- or *N*-linked glycans or glycosphingolipids [38]. Usually, *N*-linked oligosaccharides need to be treated with peptide *N*-glycosidase, while *O*-linked oligosaccharides are treated with either acid or alkali. These released oligosaccharides are then derivatized with either reductive amination in the presence of NaBH_3_CN or Michael addition [31,39]. From a structural point of view, *O*-linked oligosaccharides are relatively simpler glycans without mannose, while *N*-linked oligosaccharides are more complex, but have a principally ordered structure. Aspartylglucosaminuria is one LSD mostly found in Finland or the descendants of that area. Recently, our group identified a Japanese patient with this disorder through a combination of genetic and TOF-MS-based biochemical diagnoses [40]. From analytical point of view, *N*-linked oligosaccharides are also readily detectable without further reaction [41,42].

A recent study by Lawrence and colleagues provides a comprehensive profile of glycans in individuals with β-galactosidase (GLB) deficiency [38]. In this paper, the authors use tandem mass spectrometry and glycan reductive isotope labeling to provide evidence that GLB deficiency is not a simple ganglioside accumulation disorder, but rather it elevates many oligosaccharides including many β-linked galactoses. Specifically, they showed an elevation of a bi-branched octasaccharide with galactose at reducing end, namely A2G2′ glycan, in GLB null mice and affected individuals, respectively. Oligosaccharides are composed of hexose, hexosaminoglycan, fucose, and sialic acid, thus the expected molecular weight may be easily calculated. Based on such data and fragmentation pattern that has been already known, each oligosaccharide is to be identified using LC-MS/MS-based technique.

Sialidosis is caused by two enzymes: One involves acid neuraminidase (NEU1, EC 3.2.1.18), which cleaves the glycosyl bond between sialic acid and oligosaccharides. The other involves cathepsin A (CTSA, EC 3.4.16.5), which stabilizes both NEU1 and GLB. An earlier review summarized that the concentration of sialylated oligosaccharides in affected individuals reaches approximately 100 mg/L, while that of healthy individuals remains at 0.1–0.5 mg/L of urine [43]. A subsequent study identified several species of sialylated oligosaccharides in the urinary samples of affected individuals [42]. More recent studies have quantified sialylated oligosaccharide concentrations using LC-MS/MS [39,44]. Additionally, sialylated oligosaccharides have been postulated to play a biological role through extending the half-life of proteins. For example, Chinese hamster ovary (CHO) cells are a widely used cell line for biological production of proteins due to their high production efficiency [45]. To maximize the yield of final product, these cells may be further engineered by the overexpression of sialyltransferases and inhibition of sialidases. One study [46] found that a common phenotype in sialidosis patients and Neu1-deficient mice is hepatosplenomegaly due to extramedullary hematopoiesis. This is likely explained by the decreased retention of hematopoietic cells in bone marrow due to the enhanced secretion of proteases into extracellular fluid through secretory lysosomes. Interestingly, the latter study identified LAMP1, a lysosomal glycoprotein, as a target of the NEU1 enzyme. As expected, the half-life of LAMP1 in Neu1-deficient cells increased four times with respect to wild-type cells, as demonstrated by a pulse-chase experiment.

Pompe disease is a muscular disorder associated with a deficiency of α-glucosidase (GAA) activity [4]. In infantile-onset Pompe disease, the affected individuals may not survive beyond two years without treatment [4]. Glucose tetrasaccharide (Glc_4_) is a known biomarker for Pompe disease [47]. In a couple of studies, [^13^C]_6_-labeled Glc_4_ has been synthesized and used as the internal standard [47,48].

## 6. Enzyme Activity

The enzyme activity for LSDs has been examined in the plasma as well as the concentrate of white blood cells at the beginning. To quantify the enzyme activity, fluorogenic substrate has been widely used. A milestone discovery that some LSD enzyme activity was stable was reported by Dr Chamoles in 2001 [49]. Based on this observation, neonatal screening using dried blood spots (DBS) becomes realistic. There are several reasons behind this. Essentially, DBS can be transported by regular courier service without dry ice; thus, many samples can be collected at reasonable cost. Implementation of neonatal screening depends not only on advances in technology, but also on government policy. A concise review for newborn screening for LSDs has been published very recently [50]. In case in the United States, if a condition is to be included in the recommended uniform screen panel (RUSP), such a screening must be feasible and multiple individuals identified by screening should be presented. Currently, Pompe disease and MPS I have been listed in RUSP. Very recently, a pilot study for MPS II, IIIB, IVA, VI, and VII was completed in Washington state [51]. Thus, the number of conditions to be screened by newborn screening is expanding.

From technological point of view, as mentioned, the enzyme activity for some LSDs are stable in DBS. The assay can be done using a substrate for fluorometry or tandem mass spectrometry today. In particular, a series of studies for synthesis of substrates and internal standards for LSD enzyme assay was conducted [52]. The internal standards are deuterated; thus, the accumulating enzyme reaction products can be readily quantified based on the comparison to the known amount of internal standard. The initial set of enzyme assay contains six LSD enzymes, such as GAA for Pompe, GLA for Fabry, α-L-iduronidase for mucopolysaccharidosis type I, acid β-glucosidase for Gaucher, acid sphingomyelinase for Niemann–Pick disease type A/B, and galactosylceramidase for Krabbe disease, due to relatively high prevalence of disorders among LSDs. More recently, the number of reported combinations of substrate and internal standard are now expanding, including lysosomal acid lipase [53], MPS IIIA [54], mannosidosis [55], and fucosidosis [55], respectively.

It is well appreciated that each disorder appears to have skewed prevalence worldwide. For example, MPS II is commonly found in Asia while in other area MPS I is predominant. Based on this fact, neonatal screening for MPS II is implemented in Taiwan [56]. As reconciled with many other examples, each population has a unique distribution of genetic variant with or without pathogenesis. This idea is also applicable to MPS II where the variant and its frequency in tested population is significantly different in the US and Taiwan (Table 2). For example, the most frequently identified variant in Taiwan, is an intronic variant without any mutation in exons. In contrast, in the state of Washington, U.S., a c.851C>T (p.P284L) variant was the most frequently detected that is classified as benign. Interestingly, the same variant was identified in Taiwan, but the incidence was approximately 1/10 (Table 2). When a specimen with low enzyme activity was identified, the specimen undergoes genetic testing. In case the mutation may not be found in the database, the sample might be considered as variant of unknown significance (VUS). In most cases, alternate biomarker assay other than enzyme activity helps to discriminate whether such a specimen is true positive or not. This is effective for the multiple genetic alteration that contains VUS, because such a mutation is usually sporadic and not found in database.

## 7. Future Perspectives

LSDs include more than 50 disorders linked to the lysosomes. Most of them are caused by a deficiency of enzymes, thus the biomarkers for these disorders are generally the substrates of enzymes. For example, LysoGb_3_, HS, and oligosaccharides elevate when enzyme(s) responsible for GLA, MPS types I, II, IIIA-D or VII, and α/β-mannosidases or GAA, respectively, are defective. With advancements in analytical chemistry, especially the MS/MS technique, the methodology for some of these biomarkers, of which the concentration is normally very low, becomes available. Once the biomarker for each condition has been identified, a known concentration of standard chemicals, which are often isotopically labeled, is necessary. Presently, a limited number of internal standards are only available, thus further study will be required for the production of such analytical materials.

Accumulating evidences have suggested that the elevation of storage materials compared to healthy individuals is linked to the pathogenesis, while the severity of disorders might not be well correlated with the levels of biomarkers. This is most evident when ERT has been performed. In these cases, the concentration of elevating storage material under pathological conditions usually decreases in response to the treatment, but it fails to become healthy levels. Some biomarkers, such as Glc_4_ for Pompe disease, are known to be less specific. Thus, from a monitoring point of view, exploration to find better biomarkers has been beneficial for several LSDs. In fact, in Gaucher disease, plasma chitotriosidase activity and a chemokine CCL18 are known biomarkers that decrease at nearly baseline levels in response to enzyme replacement therapy [58].

Finally, apart from disorders associated with lysosomal enzyme activity, some disorders are caused by pathogenic alteration of lysosome-related proteins with no enzyme activity. The best-known example is NPC, which is caused by either the *NPC1* or *NPC2* genes. Today, multiple biomarkers for NPC have been characterized, including oxysterols, LysoSM, a variety of bile acid derivatives, and PPCS. Intriguingly, the biosynthesis of them is largely unknown. This also needs to be explored in future studies.

In conclusion, the recent advances in mass spectrometry in LSD research play an essential role in the quantification and identification of biomarkers, including small compounds and enzyme activities. At least in part, this depends on the robustness of mass spectrometric assay, which has a high sensitivity and reproducibility, and the multiplex measurement of analytes. One notable example is the capacity of this technique to determine several LSD enzyme activities within one or two minutes during newborn screening. The development of novel therapies involving enzyme replacement therapy are dependent on accurate and sensitive in vitro diagnostics. Similar innovation may take place when a novel therapy including gene therapy will be developed.

## Figures and Tables

**Table 1:** Novel biomarker for Mucopolysaccharidosis (MPS) III disease subtype (Ref [31]).

MPS III Disease-Subtype	OMIM	Enzyme	Abbreviation	Biomarker	Number of Sulfate	Other MPS
MPS IIIA	252900	*N*-sulfoglucosamine sulfohydrolase	SGSH	HN-UA	1	Not reported
MPS IIIB	252920	*N*-acetyl-α-glucosaminidase	NAGLU	(HNAc-UA)_2_	1	Not reported
MPS IIIC	252930	Heparan-α-glucosaminide *N*-acetyltransferase	HGSNAT	(HN-UA)_2_-NAc	2	Not reported
MPS IIID	252940	Glucosamine (*N*-acetyl)-6-sulfatase	GNS	(HNAc-UA)_2_	2	IVA, VI
				HNAc	1	IVA, VI

H, hexose; N, hexosamine; Ac, acetyl; S, sulfate; UA, uronic acid.

**Table 2:** The *IDS* mutations identified by neonatal screening.

Scott CR et al. (2019) (*n* = 105,214) [51]					Chuang C-K et al. (2018) (*n* = 153,032) [57]				
cDNA	Protein	Interpretation	*n*	Frequency	cDNA	Protein	GAG	*n*	Frequency
				(1/million)					(1/million)
c.851C>T	p.P284L	Benign	7	66.5	c.103+34_56dup	Intronic mutation	Negative	49	320.2
c.1090C>T	p.P364S	Benign	2	19.0	c.1499C>T	p.T500I	Negative	10	65.3
c.1499C>T	p.T500I	Benign	1	9.5	c.301C>T	p.R101C	Negative	7	45.7
c.684A>G/ c.851C>T	p.P228P+ p.P284L	Benign	1	9.5	c.1478G>A	p.R493H	Negative	4	26.1
c.1409C>T	p.S470L	At risk	1	9.5	c.103+34_56dup/ c.851C>T	p.P284L	Negative	1	6.5
c.159T>C	p.C53W	At risk	1	9.5	c.103+34_56dup/ c.851C>T/ c.1180+184T>C	p.P284L	Negative	1	6.5
c.817C>T	p.R273W	Affected	1	9.5	c.589C>T	p.P197S	Negative	1	6.5
					c.851C>T	p.P284L	Negative	1	6.5
					c.890G>A	p.R297H	Negative	1	6.5
					c.1025A>G	p.H342R	Positive	1	6.5
					c.311A>T	p.D104V	Positive	1	6.5
					c.817C>T	p.R273W	Positive	1	6.5
